# BOLD in Action: Partnering to Provide Community-based Dementia Education in Texas

**DOI:** 10.1093/geroni/igaf122.3550

**Published:** 2025-12-31

**Authors:** Andrew Crocker, Rachel Brauner, Bailey Young, Jenna Perales, Ryan Williams

**Affiliations:** Texas A&M AgriLife Extension Service, Amarillo, Texas, United States; Texas A&M AgriLife Extension Service, College Station, Texas, United States; West Texas A&M University, Canyon, Texas, United States; Alzheimer’s Association, Fort Worth, Texas, United States; Texas Tech University, Amarillo, Texas, United States

## Abstract

Due to dementia’s impact on public health, the Building Our Largest Dementia Infrastructure for Alzheimer’s Act (BOLD) directs the United States Centers for Disease Control and Prevention to enhance public health infrastructure for dementia risk reduction, early detection, and diagnosis. As a recipient of BOLD funding, the Texas Department of State Health Services (DSHS) embraced the effort in its four-year state plan for Alzheimer’s disease to, in part, “leverage existing and new partnerships to provide education to the public and healthcare professionals through community education events and awareness campaigns.” In 2024, Texas A&M AgriLife Extension Service, the community-based education and outreach arm of the Texas A&M University System, partnered with DSHS and Alzheimer’s Association Chapters across Texas to implement three hour-long programs to help improve dementia literacy and reduce controllable risk factors. In this exploratory research (IRB # 2024-0796), we seek to determine the effectiveness of the educational programming as well as any nuance in the results related to age, residing in a rural area, and residing in a medically underserved area. This study analyzes pre-post evaluation data from three community-based programs: 10 Warning Signs of Alzheimer’s Disease, Healthy Living for Your Brain and Body, and Understanding Alzheimer’s and Dementia. Results reveal increase in knowledge with mixed patterns across groups, highlighting both similarities and notable differences based on demographics and residency. Findings from this research help reinforce the need for this type of inter-agency partnership and the resulting community-based education for helping improve dementia literacy and mitigating risk factors.

